# The Effect of Adding a Smartphone-Based Platform to the Metabolic Bariatric Surgery Nutritional Preparation Process: A Randomized Controlled Trial

**DOI:** 10.1007/s11695-025-07732-9

**Published:** 2025-03-12

**Authors:** Yafit Kessler, Mona Boaz, Limor Mardy-Tilbor, Asnat Raziel, Nasser Sakran, David Goitein, Andrei Keidar, Hasan Kais, Bella Azaria, Shiri Sherf-Dagan

**Affiliations:** 1 Department of Nutrition Sciences, Ariel University, Ariel, Israel; 2 Department of Nutrition, Assuta Medical Center, Tel Aviv, Israel; 3 Assia Medical Group, Assuta Medical Center, Tel Aviv, Israel; 4 Assuta Bariatric Center, Assuta Hospitals, Tel Aviv, Israel; 5 Department of Surgery, Holy Family Hospital, Nazareth, Israel; 6 The Azrieli Faculty of Medicine Safed, Bar-Ilan University, Ramat Gan, Israel; 7 School of Medicine, Faculty of Medicine and Health Sciences, Tel-Aviv University, Tel-Aviv, Israel; 8 Department of General Surgery, Sheba Medical Center, Ramat Gan, Israel; 9 Division of General Surgery, Sourasky Medical Center, Tel Aviv, Israel; 10 Division of Surgery, Shamir Medical Center, Zerifin, Israel; 11 Medicine Division, Assuta Medical Center, Tel Aviv, Israel

**Keywords:** Metabolic bariatric surgery, Preparation process, Registered dietitian, Health education, EHealth, Mobile application

## Abstract

**Background:**

Metabolic bariatric surgery (MBS) candidates undergo a comprehensive nutritional preparation process by a registered dietitian (RD). The effect of eHealth interventions on the MBS preparation process is unknown.

**Objectives:**

To assess the impact of adding an application to the nutritional preparation process on pre-surgery nutritional knowledge, physical, and behavioral parameters among MBS candidates.

**Methods:**

An open-label randomized controlled trial among MBS candidates. All participants received 3–6 meetings with an RD and the intervention group also received access to an application containing information modules and a communication platform. Data was collected at baseline and end of preparation.

**Results:**

Forty participants were recruited, of them 67.5% women, with a mean age and body mass index of 34 ± 10.1 years and 43.5 ± 6.0 kg/m^2^, respectively. Nutritional knowledge, anthropometrics, functionality, adherence to most behavioral recommendations, and subjective state of health improved in both groups (*P* Time ≤ 0.044). Physical activity initiation (i.e., beginning of regular exercise engagement) was higher among the intervention group (40% at baseline and 68% at end of preparation vs 35% at baseline and 32% at end of preparation for interventions and controls, respectively, *P* Time × Group = 0.026). The application was rated as providing added value (8.2 on a scale of 1 (no added value) to 10 (meaningful added value)).

**Conclusions:**

Nutrition preparation process with an RD improved MBS knowledge, adherence to behavioral recommendations, subjective state of health, and modestly enhanced weight and functionality outcomes among MBS candidates. Although rated as having an added value, incorporating an application had only a minimal impact on these outcomes.

**Supplementary Information:**

The online version contains supplementary material available at 10.1007/s11695-025-07732-9.

## Introduction

Obesity is a complex chronic relapsing disease with global health consequences [1, 2]. Metabolic bariatric surgery (MBS) is currently the most effective treatment for weight loss and adiposity-related complications improvement among participants with severe obesity [3]. All MBS candidates are required to undergo a comprehensive medical, nutritional, and psychological assessment by a multidisciplinary team to evaluate their suitability and readiness for the surgery [3]. A qualified registered dietitian (RD) conducts the pre-surgical nutritional evaluation and preparation, which involves acquiring essential knowledge and adapting the required eating habits following surgery, such as chewing slowly, separating solids from liquids, prioritizing proteins in most meals, addressing nutritional deficiencies, managing glycemic control, and tailoring a personalized weight-loss program [4, 5]. Mobile technologies can provide an easy-to-use and cost-effective platform to educate, engage, and intervene and may be utilized to increase patient engagement [6], though they are underutilized in MBS, and studies investigating the effect of their integration in the nutritional and lifestyle preparation process compared to standard care are limited [7, 8]. The use of smartphone-based platforms to improve medical outcomes has been demonstrated in various fields of medicine [9]. Therefore, this study aimed to evaluate the impact of integrating a smartphone-based platform into the MBS nutritional preparation process on pre-surgery nutritional knowledge, physical, and behavioral parameters among MBS candidates.

## Methods

### Patient Eligibility and Enrollment

**Fig. 1:**
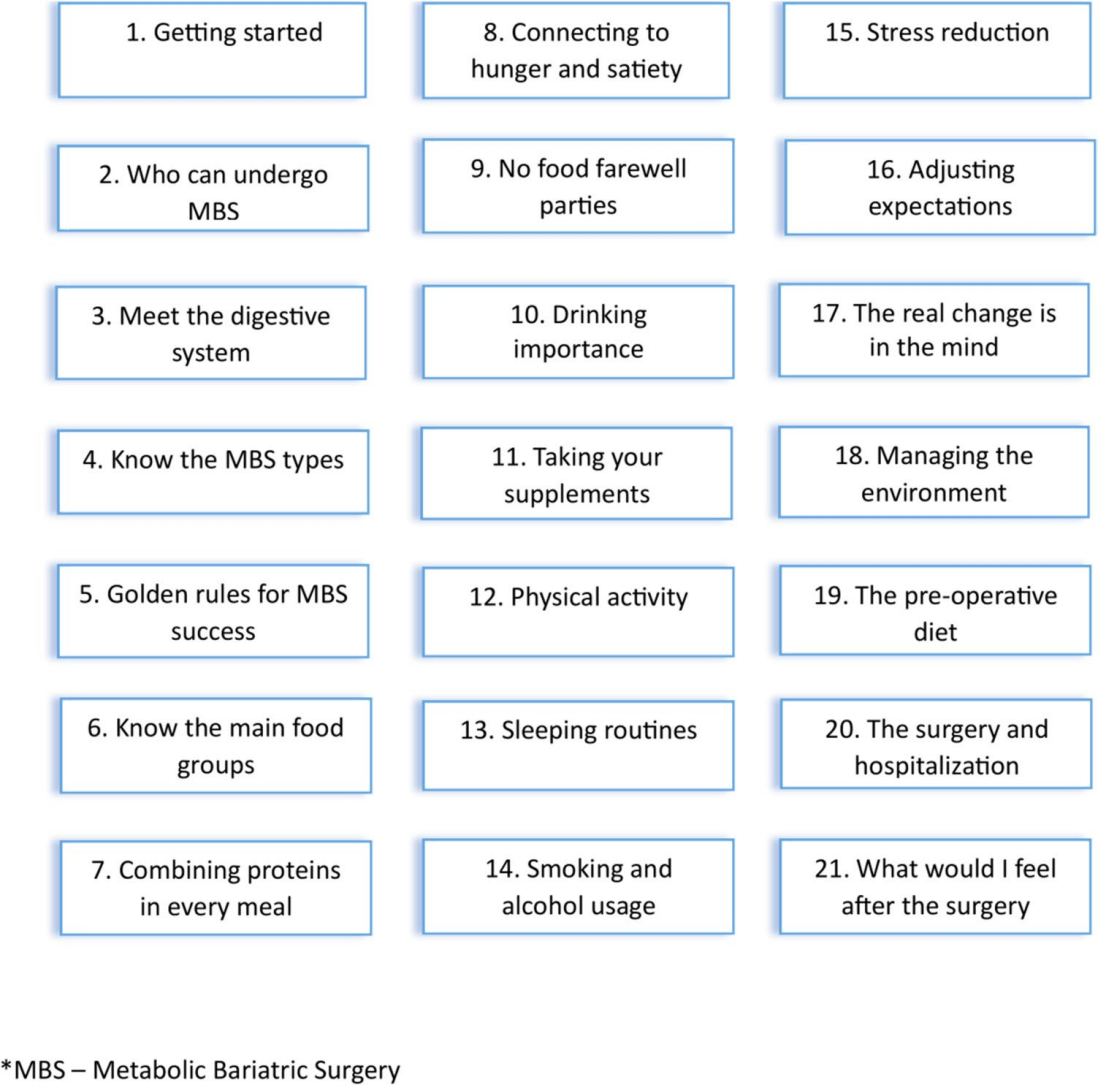
Study application modules

An open-label parallel randomized controlled trial (RCT) was conducted. Participants were recruited while attending MBS clinics at Assuta Medical Centers and through a dedicated advertisement on social media networks. After signing informed consent, participants were randomly assigned to intervention or control groups in a 1:1 ratio stratifying for gender. Inclusion criteria included age ≥ 18 years, body mass index (BMI) ≥ 40 kg/m^2^ or BMI ≥ 35 kg/m^2^ with coexisting adiposity-related complications; planned primary sleeve gastrectomy (SG), Roux-en-Y gastric bypass (RYGB), or one anastomosis gastric bypass (OAGB); ability to read and speak Hebrew; and owning a smartphone. Exclusion criteria were previous MBS; more than one MBS preparation meeting with an RD before study recruitment; contraindications for MBS (e.g., active addiction to alcohol or uncontrolled psychiatric disorders); and insulin-treated diabetes. All participants were scheduled for 3–6 face-to-face appointments with 1–3 weeks intervals between meetings for nutritional assessment and preparation with an RD as mandated for MBS candidates in Israel [10], while each meeting was scheduled for 30–45 min. The duration of preparation and the number of meetings were determined individually for each patient based on clinical findings and progress through the preparation stages. The meetings encompassed essential nutritional evaluations, including anthropometric measurements, assessment of eating and lifestyle behaviors, identification of nutritional deficiencies, and evaluation of MBS knowledge and expectations. They also covered nutritional preparation elements, such as enhancing MBS knowledge, adapting recommended eating and lifestyle behaviors, ensuring compliance with the recommended supplementation, and initiating physical activity (PA). A detailed description of these components is provided in Supplementary Table 1 [5]. Additionally, the intervention group received access to the study’s smartphone application, developed for the research by “Refeed” (i.e., an online platform for accompanying patient’s journey in diseases with nutritional treatment-based protocol). The study’s application was comprised of text and video-based modules delivering information customized for MBS candidates including the importance of proteins, supplementation usage, the pre-operative diet, behavioral rules to maximize success, and managing the environment after the surgery, with new content made available daily for a continuous period of 21 days, alongside a communication platform to ask questions and receive answers from an RD. A detailed list of the delivered content is presented in Fig. 1. All participants were informed of their assigned randomization group at the end of the first preparation meeting with the RD following baseline data collection.

The study protocol was approved by the ethics committee of the institutional review board of Assuta Medical Centers (#0018–20-ASMC). The study was preregistered on the NIH registration website (TRIAL no. NCT04451499). The study methods were based on the Consolidated Standards of Reporting Trials (CONSORT) statement [11].

### Baseline and Follow-up Evaluations

Data was collected at the first and last meeting with the study’s RD. Demographics and medical information were collected at the first meeting by interviews. Comorbidity was defined by a physician’s diagnosis in medical files, abnormal blood tests, or use of designated medications. All other measurements were collected at baseline and post-intervention.

#### Primary Outcome

##### MBS Nutritional Knowledge

Participants were asked to answer an MBS nutritional knowledge questionnaire which was previously created based on a literature review and professional experience and overwent a process of face, content, and construct validity. The questionnaire score was calculated on a 0–100-point scale, with higher scores indicating greater MBS nutritional knowledge [12]. Due to updates in supplementation routines and the availability of products in Israel, we have revised the correct answer for the supplementation question (Q5) to include “multivitamin” and “vitamin D” (choosing “calcium” or “vitamin B12” was not considered incorrect and did not affect the score).

#### Secondary Outcomes

##### Anthropometrics and Functionality

Weight was measured on a digital scale, height was measured on an altimeter, and BMI was calculated afterward. Waist circumference (WC) was measured twice at the level of the umbilicus by a measuring tape and an average was calculated [13]. Body composition was measured using multi-frequency bioelectrical impedance analysis (Inbody370S®, InBody Co., Ltd.) according to the manufacturer’s standard specifications. Cut-off points for fat mass percentage were used to define obesity (≥ 25.0% for men and ≥ 35.0% for women) as recommended [14]. Static muscle strength of the upper extremities was measured by using a digital handgrip dynamometer (Jamar plus digital). Participants were instructed to align their elbows at a 90° angle while seated and to squeeze the handle to exert maximum force using their dominant hand. Three measurements were recorded with a 15-second break between each, and an average was calculated [15].

##### Exercise and Physical Activity (PA)

The level of exercise was evaluated subjectively by questioning participants about their exercise routine over the last month, including type, frequency, and duration of each exercise, and classifying them according to the achievement of at least 150 min/week of aerobic activity [16]. Objective measurement of PA was made by pedometers (OMRON Step Counter WS One) given to participants at the first meeting. Step counts were recorded for seven consecutive days after the first and last meeting with the RD. If fewer than 3 days of data were recorded, the measurements were considered missing data and were not included.

##### Compliance with the MBS Nutritional and Lifestyle Recommendations

Compliance was assessed by questioning participants about following the key recommended eating behaviors in the last month (no/partially/always). The key recommended eating behaviors asked included separating liquids from solids, avoiding carbonated drinks, chewing food slowly and thoroughly, dividing food intake into 4–6 meals throughout the day, ending meals when feeling “comfortably full,” consuming high-protein foods in most meals, and preferring to eat solid food items (e.g., boiled egg, chicken breast, salad) over crunchy or liquid food items (e.g., ice cream, crackers, cookies, cakes) in most meals. These key items were based on well-established clinical practice recommendations [17].

##### Hydration State

Hydration state was evaluated objectively by collecting spot urine samples in opaque containers and categorizing them based on a standardized urine color scale ranging from 1 (pale yellow, indicating diluted urine) to 8 (dark brown, indicating concentrated urine), with 4 or above set as a cut-off for dehydration [18].

##### Perception of State of Health

Perception of state of health was assessed by asking participants to rate their overall state of health on a visual analog scale (VAS) ranging from 0 (reflects the “worst imaginable state of health”) to 100 (reflects the “best imaginable state of health”) [19].

#### Study’s Application Rating

At the end of the intervention, participants from the intervention group were asked regarding the “easiness of usage of the app” on a scale of 1 (“very easy”) to 10 (“very difficult”) and the “effectiveness of app” on a scale of 1 (“no added value”) to 10 (“meaningful added value”).

##### Statistical Analyses

were conducted by SPSS statistical package, version 29. Descriptive statistics were used to describe the distribution of variables associated with the characteristics of the study sample. Continuous variables were presented as means ± standard deviation (SD) or median (interquartile range, IQR), and dichotomous/categorical variables as proportions. The normality of the distribution for continuous variables was tested by several pathways including histogram, Kolmogorov–Smirnov test, and Q-Q plot. If normality was rejected, non-parametric tests were used. For comparisons of continuous variables between groups, the independent sample *t*-test or Mann–Whitney test was utilized, as needed. For comparisons of dichotomous or categorical variables between groups, the chi-square test or Fisher’s exact test was utilized, as needed. Linear mixed model repeated measures analysis and generalized estimating equations repeated measures analysis were conducted to test differences in continuous variables and dichotomous variables within and between groups over time, respectively. To compare dichotomous data within groups over time, the McNemar test was used. Analysis was performed adhering to the intention-to-treat principle. The level of significance for all analyses was set at *P* < 0.05.

Sample size was calculated in the G*power software (version 3.1.9.4) to detect a difference of 12 points with a SD of 13.4 in the MBS nutritional knowledge questionnaire score between groups [12], while considering 80% power, a 0.05 one-sided *α* level, a 1:1 ratio between the study groups, and 10% attrition rate. A total sample size of 38 participants was calculated. Therefore, 20 participants were recruited for each group.

## Results

### Characteristics of Study Participants

**Fig. 2:**
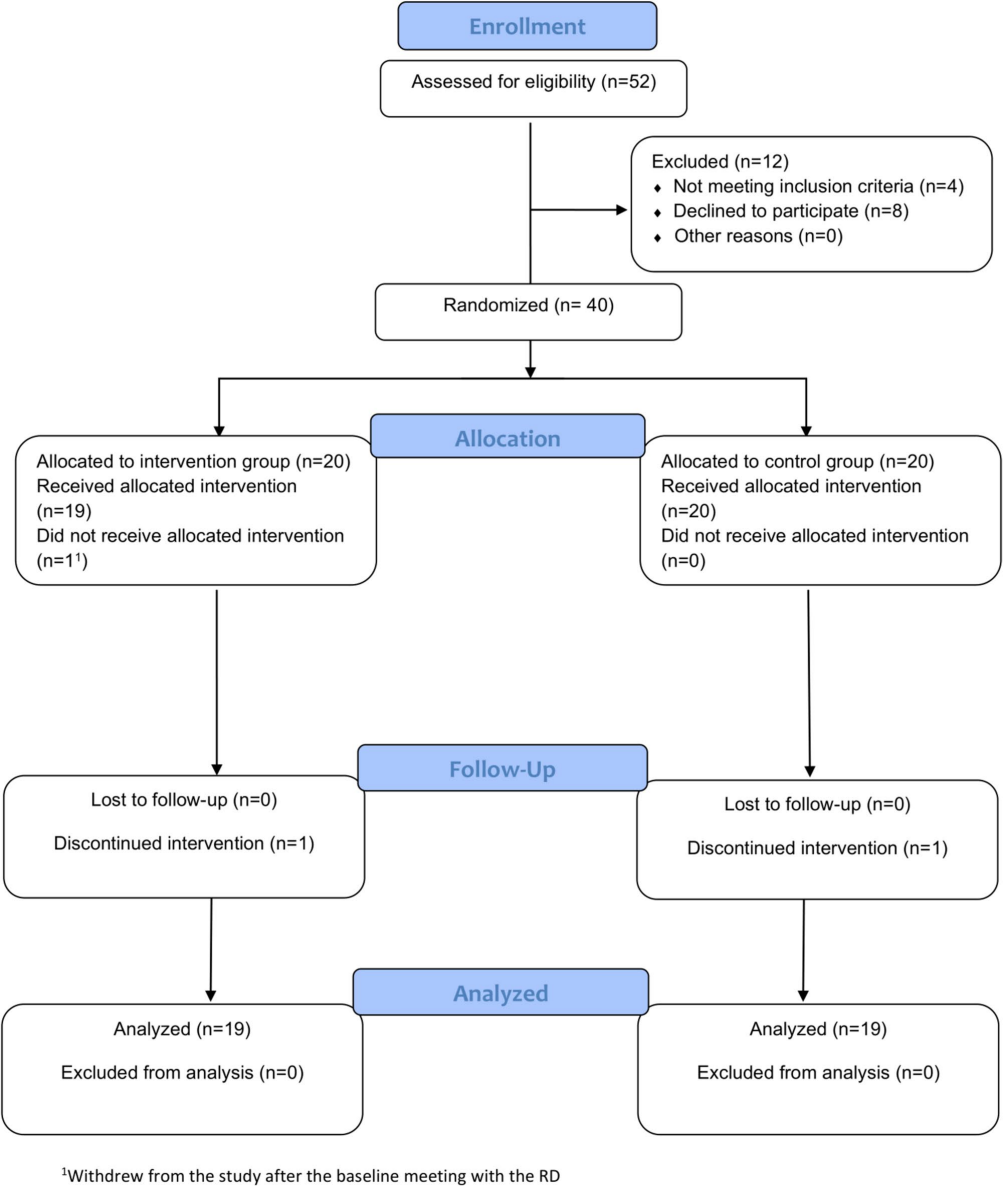
A flow chart of the study’s participants

A total of 40 MBS candidates (67.5% females) with mean age and BMI of 34.0 ± 10.1 years and 43.5 ± 6.0 kg/m^2^ were recruited to the study and randomized into the intervention group (*n* = 20) or control group (*n* = 20). Of them, 2 participants (*n* = 1 in each group) decided to give up the surgery and discontinued the preparation process, and 38 participants (95%) completed the study; a flow chart of the study’s participants is presented in Fig. 2; 9 of the 19 participants in the intervention group who completed the study viewed all 21 application modules (47.4%), 9 viewed ≤ 13 modules (47.4%), and 1 did not view any modules. Interestingly, only one participant used the communication platform offered through the application to ask questions regarding diet choices.

Table 1 displays the baseline characteristics of the participants. There were no significant differences in baseline characteristics between the groups. The median (IQR) number of preparation meetings with the RD during the study period was similar between groups (6 (3,6) and 4.5 (3,6) for the intervention and control group, respectively, *P* = 0.414).

**Table 1:** Baseline characteristics of study groups

Parameter^1^	All population (*n* = 40)	Intervention group (*n* = 20)	Control group (*n* = 20)	*P* value for between-group difference
**Demographics**				
Age (years)	34.0 ± 10.2	34.6 ± 9.8	33.4 ± 10.8	0.704
Gender (% female)	27 (67.5%)	13 (65.0%)	14 (70.0%)	0.736
Marital status (% married)	23 (57.5%)	10 (50.0%)	13 (65.0%)	0.161
Education (% with > 12 years)	23 (57.5%)	10 (50.0%)	13 (65.0%)	0.337
**Anthropometrics**				
Weight (kg)	123.3 ± 27.3	128.2 ± 34.4	118.4 ± 17.1	0.265
Height (meter)	1.68 ± 0.11	1.69 ± 0.13	1.67 ± 0.07	0.547
BMI (kg/m^2^)	43.5 ± 6.1	44.4 ± 7.4	42.6 ± 4.5	0.349
**Health status**				
Type 2 diabetes (n,%)	4 (10.3%)	1 (5.3%)	3 (15.0%)	0.605
Dyslipidemia (n,%)	19 (48.7%)	6 (31.6%)	13 (65.0%)	**0.037**
Current smokers (n,%)	3 (7.5%)	1 (5.0%)	2 (10.0%)	1.000
**Nutritional deficiencies**^2^				
Any deficiencies (n,%)^3^	33 (86.8%)	17 (89.5%)	16 (84.2%)	1.000
Anemia (n,%)^3^	5 (13.2%)	3 (15.8%)	2 (10.5%)	1.000
Iron (n,%)^3^	10 (26.3%)	4 (21.1%)	6 (31.6%)	0.461
Folate (n,%)^4^	14 (37.8%)	7 (38.9%)	7 (36.8%)	0.898
Vitamin B12 (n,%)^3^	2 (5.3%)	2 (10.5%)	0 (0.0%)	**< 0.001**
Vitamin D (n,%)^3,5^	31 (81.6%)	17 (89.5%)	14 (73.7%)	0.405
Number of dietitian appointments (median, IQR)	5 (3,6)	6 (3,6)	4.5 (3,6)	0.414

Abbreviations: *BMI* body mass index, *IQR* inter quartile range

^1^All outcomes are presented as mean ± SD or as n (percentages), unless otherwise stated

^2^Micronutrient deficiency was defined as a serum level below the reference range recommended per health maintenance organization (HMO)

^3^For this outcome *n* = 38 (*n* = 19 and *n* = 19 for the intervention and control group, respectively)

^4^For this outcome *n* = 37 (*n* = 18 and *n* = 19 for the intervention and control group, respectively)

^5^Vitamin D insufficiency levels were defined as < 30 ng/ml

#### Primary Outcome

##### MBS Nutritional Knowledge

Similar trends were observed between the groups over time for the mean of MBS nutritional knowledge questionnaire scores (*P* Time × Group = 0.766), while in both groups, the mean scores exhibited a significant increase post-intervention compared to baseline (*P* × Time < 0.001) (Table 2).

**Table 2:** Changes in nutritional knowledge, perception of state of health, anthropometrics, functionality, exercise and physical activity, and hydration in the intervention and control groups over time

Outcome variable^1^	Group^a^	Baseline	Post-intervention	*P* Time^2^	*P* Group^3^	*P* Time × Group^4^
MBS nutritional knowledge						
Nutrition knowledge score	Intervention	68.4 ± 3.7	85.1 ± 2.0^b^	** < 0.001**	0.077	0.766
	Controls	61.2 ± 3.6	79.1 ± 2.0^b^			
Perception of state of health						
Perception of state of health (VAS)	Intervention	49.7 ± 5.7	62.1 ± 5.1^b^	**0.031**	0.876	0.288
	Controls	54.7 ± 5.6	59.1 ± 5.1			
Anthropometrics and functionality						
Weight (kg)	Intervention	128.2 ± 6.1	127.0 ± 5.8^b^	**0.034**	0.266	0.459
	Controls	118.4 ± 6.1	117.8 ± 5.8			
BMI (kg/m^2^)	Intervention	44.4 ± 1.4	44.1 ± 1.3	0.063	0.356	0.623
	Controls	42.6 ± 1.4	42.4 ± 1.3			
WC (cm)	Intervention	131.4 ± 3.5	129.8 ± 3.4	0.064	0.362	0.371
	Controls	126.4 ± 3.5	125.8 ± 3.4			
FM (kg)	Intervention	62.5 ± 3.0	61.8 ± 2.9	0.348	0.397	0.310
	Controls	58.6 ± 3.0	58.6 ± 2.9			
FM (%)	Intervention	49.2 ± 1.0	49.2 ± 1.0	0.767	0.806	0.609
	Controls	49.5 ± 1.0	50.0 ± 1.0			
FFM (kg)	Intervention	65.6 ± 3.6	65.2 ± 3.6	0.166	0.251	0.843
	Controls	59.6 ± 3.6	59.3 ± 3.6			
SMM (kg)	Intervention	37.0 ± 2.2	36.8 ± 2.1	0.332	0.257	0.887
	Controls	33.5 ± 2.2	33.3 ± 2.1			
Handgrip (kg)	Intervention	30.8 ± 2.9	32.1 ± 2.7	**0.044**	1.000	0.646
	Controls	31.0 ± 2.9	31.9 ± 2.7			
Exercise and physical activity						
PA (% who answered yes)	Intervention	40	68^b^	0.088	0.147	**0.026**
	Controls	35	32			
PA ≥ 150 min aerobic activity per week (%)	Intervention	5.0	10.5	0.156	1.000	1.000
	Controls	5.0	10.5			
Average daily steps^5^	Intervention	4302.1 ± 527.3	4543.7 ± 617.4	0.814	0.287	0.348
	Controls	3834.0 ± 484.0	3432.7 ± 633.9			
Hydration status						
Hydration scale^6,7^	Intervention	4.9 ± 0.4	4.3 ± 3.6	0.307	0.245	0.237
	Controls	5.1 ± 0.4	5.1 ± 0.4			

Abbreviations: *BMI* body mass index, *FFM* fat free mass, *FM* fat mass, *MBS* metabolic bariatric surgery, *PA* physical activity, *SMM* skeletal muscle mass, *VAS* visual analog score, *WC* waist circumference

^1^Data are presented as estimated mean (± SE) according to the mixed model analysis

^2^*P* Time = *P* value for changes over time in the two groups

^3^*P* Groups = *P* value for between-groups differences over time

^4^*P* Time × Group = *P* value for interaction between the trend of change over time and the group effect

^5^For this outcome *n* = 35 (*n* = 16 and *n* = 19 for the intervention and control group, respectively)

^6^For this outcome *n* = 38 (*n* = 19 and *n* = 19 for the intervention and control group, respectively)

^7^Based on a standardized urine color scale (1 = pale yellow to 8 = dark brown); 4 or above was considered as cut-off for dehydration

^a^No between group differences were found at baseline for any outcome variable (*p* ≤ 0.05)

^b^Within group differences compared to baseline (*p* ≤ 0.05)

#### Secondary Outcomes

##### Anthropometrics and Functionality

Similar trends were observed between the groups over time for mean weight status and handgrip muscle strength results (*P* Time × Group = 0.459 and 0.646, respectively), while a modest reduction in weight and a modest increase in handgrip muscle strength results were observed post-intervention compared to baseline for both groups (*P* × Time = 0.034 and 0.044) (Table 2).

##### Exercise and PA

Similar trends were observed between the groups over time for the percent of participants achieving the recommended goal for PA ≥ 150 min of aerobic activity per week (*P* Time × Group = 1.000), with the majority in both groups not reaching the recommended goal. Interestingly, different trends were observed between the groups over time for the percent of participants performing any PA (*P* Time × Group = 0.026), as a significant increase post-intervention was observed only within the intervention group (40 vs. 68% at baseline and post-intervention, respectively, *P* = 0.005) (Table 2).

##### Compliance with the MBS Nutritional and Lifestyle Recommendations

**Fig. 3:**
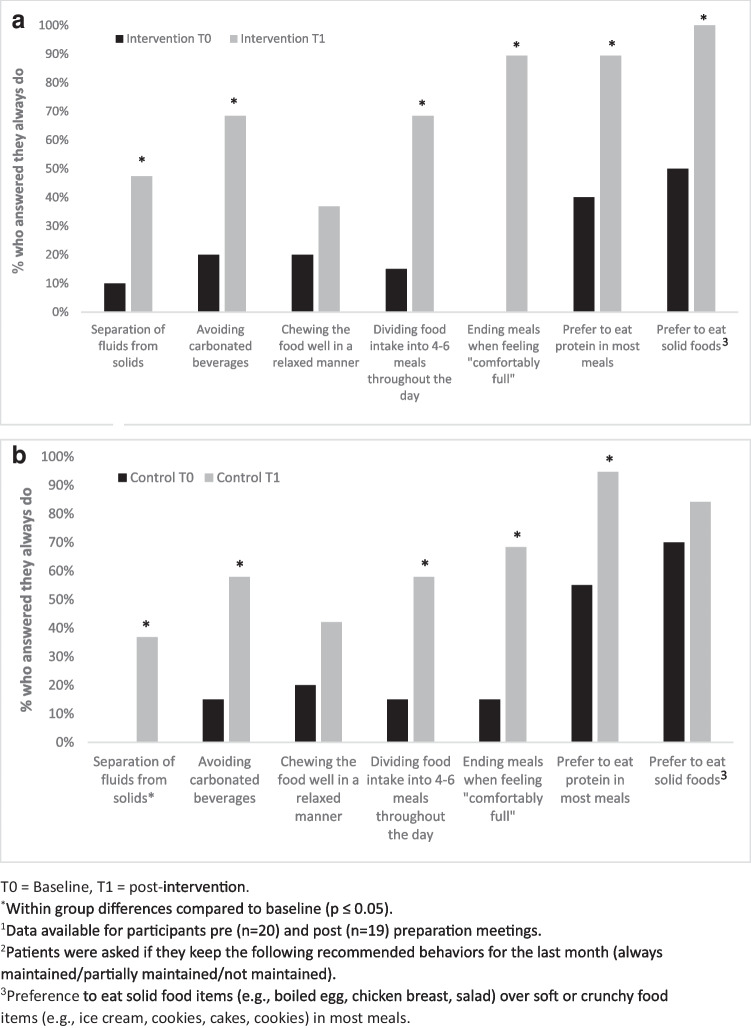
Differences in eating behaviors pre- and post-preparation in the intervention group (**a**) and in the control group (**b**)^1,2^

**Fig. 4:**
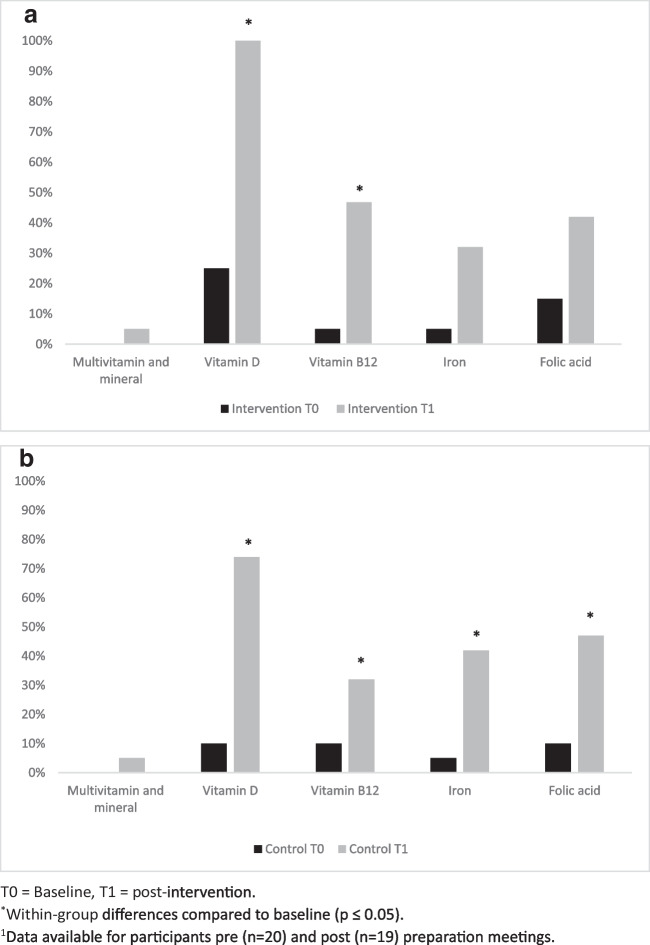
Nutritional supplementation usage pre- and post-preparation in the intervention group (**a**) and in the control group (**b**)^1^

An improvement in adherence to most recommended eating behaviors and an increase in supplementation usage was seen in both groups post-intervention compared to baseline (Fig. 3 and Fig. 4, respectively).

Similar trends were observed between the groups over time for hydration status by mean urine color scale (*P* Time × Group = 0.237) and for perception of state of health by VAS scores (*P* Time × Group = 0.288). Notably, VAS scores increased significantly only within the intervention group from baseline to post-intervention (49.7 ± 5.7 vs. 62.1 ± 5.1, respectively, *P* = 0.026) (Table 2).

##### Study’s Application Rating

The application was rated by the intervention group as easy to use; 1.8 on a scale of 1 (“very easy”) to 10 (“very difficult”), and as providing added value; 8.2 on a scale of 1 (“no added value”) to 10 (“meaningful added value”).

## Discussion

The growing digitalization worldwide entails an opportunity to implement eHealth interventions into the MBS nutritional and lifestyle preparation process [20, 21]. Such integrations may offer a reliable means of delivering information to a targeted population that often resorts to social media, where information may be misleading or inaccurate [22]. Despite the well-known importance of the pre-surgical nutritional preparation process, consensus on a standardized nutritional education method for MBS candidates is currently lacking, contributing to substantial variation in practices among different centers [23]. Moreover, information regarding the feasibility, effectiveness, type, and duration of an appropriate preoperative diet regimen is presently scarce and controversial [24]. The present study aimed to evaluate the effect of adding a smartphone-based platform into the MBS nutritional preparation process while examining pre-surgery nutritional knowledge, physical, and behavioral parameters among MBS candidates. Although participants rated the application as providing added value and being easy to use, integrating a smartphone-based platform into the preparation process resulted mainly in a significant increase in PA initiation, but did not yield any additional significant advantages over the control group in other outcomes. Still, this finding holds clinical significance as initiating and enhancing PA before MBS has beneficial health value and may improve cardiorespiratory fitness [25].

Despite the modest impact of the intervention in the present study, both groups demonstrated improvements in MBS-related nutritional knowledge, adherence to most eating and lifestyle recommendations, and perception of the state of health. Additionally, both groups experienced slight weight reduction and improvement in handgrip muscle strength following the nutritional preparation process. These findings reinforce the importance and emphasize the benefits of nutritional preparation process with an RD for MBS candidates. Improving MBS nutritional knowledge and reinforcing behavioral changes before MBS may benefit patients following MBS by enhancing adherence to essential dietary behaviors and supplementation regimens. This, in turn, has the potential to mitigate post-surgery-related complications such as nutritional deficiencies and loss of muscle and bone mass, as well as prevent common gastrointestinal symptoms related to inadequate eating behaviors [26].

Implementing desired behavioral changes pre-surgery enables patients to reach the surgery in better nutritional status, which is recommended by the leading international MBS organizations [3] and may potentially contribute to better weight outcomes [27].

As noted, both groups demonstrated a modest weight reduction and handgrip muscle strength improvement throughout the nutritional preparation process. Nevertheless, these changes do not hold clinical significance. A plausible explanation for these less-than-expected results might be the short intervention time. At present, international MBS organizations recommend engaging in pre-operative weight loss efforts as part of the preparation process [24]. Nevertheless, pre-surgery weight loss necessity is questionable, and its post-surgery benefits are currently debatable [28].

While there is inherent potential for combining applications within the MBS preparation process, only a limited number of studies have delved into this topic so far. Our result is similar to a recently published RCT investigating the effect of an application program vs. usual care among 50 MBS candidates on weight, dietary intake, and PA outcomes before the surgery, which showed no significant differences for BMI and calorie intake, but higher maintenance of PA level for the app group along an 8-week intervention [8]. On the contrary, a small prospective study among 20 MBS candidates receiving a smartphone app in addition to the MBS preparation process resulted in significant eating habits and PA changes at the end of the intervention, although this study had a longer intervention time of approximately 12 weeks and did not have a control group [6].

The acceptance and utility of implementing apps into the MBS process have been reported among MBS patients. Integrating eHealth strategies during the MBS path seems to be valuable from the patient’s perspective. Indeed, in the present study, the application was rated as easy to use and as providing high-added value by the participants in the intervention group. According to a systematic review investigating the effectiveness of eHealth strategies for MBS participants, their implementation in the preparation process can assist MBS candidates in acquiring health information and knowledge and improving lifestyle habits [21]. Moreover, allowing participants access to a well-accepted, easy-to-use, and reliable app can increase their motivation to educate themselves in a secure and non-judgmental setting, consequently improving their readiness for surgery and supporting health behavior change, according to studies regarding elective surgical populations [20]. In accordance with this arising need, several apps designated for MBS participants were developed and launched during the last few years, most of them combining information, support, and tracking features by allied health professionals [29]. Integrating an app into the MBS preparation process could enhance patient engagement by providing continuous access to information, thus making the preparation feel more integrated into their daily lives. The app’s visual elements, such as videos and images, might be more memorable than verbal explanations, offering additional benefits over face-to-face meetings. In addition, expanding the app’s role from a static information platform to a more interactive tool could further enhance its effectiveness in the preparation process. Future large-scale studies including tailor-made apps for MBS participants containing both education and support platforms are needed to further improve and maximize MBS patient care.

### Strengths and Limitations of the Study

This study possesses several notable strengths. First, our study is innovative and enriches knowledge in an unexplored field of incorporating tailored applications into the MBS nutrition preparation process. Second, the study employed a robust RCT methodology and maintained a high percentage of participants who completed the study. Third, parameters beyond weight loss were tested as the main outcomes of the intervention, highlighting the significance of enhancing patient’s knowledge and fostering behavioral changes during the MBS preparation process [5], which have the potential to mitigate post-MBS-related complications. Finally, the characteristics of our patients, including age and gender proportions, aligned with those in the national bariatric registry [30], demonstrating high external validity. Nevertheless, there are some limitations to the study. First, the sample size was relatively small; thus, the study may be underpowered to demonstrate statistically significant results. Second, there were relatively lower than expected “per protocol” rates of full intervention exposure to all application modules, although the majority viewed at least half of the modules. Third, social desirability bias must be taken into consideration due to the nature of the study and data collection. However, efforts were made to overcome this bias including the use of uniformed questionnaires, a single RD that conducted the research, and explaining to the participants the importance of reporting reliable data. Lastly, feedback from the intervention group on the application was limited to quantitative ratings, without accompanying qualitative insights.

## Conclusions

The nutritional preparation process with an RD has significant positive effects including improvement in patients’ MBS nutritional knowledge, enhanced adherence to eating and lifestyle recommendations and supplementation intake, better perception of state of health, and a modest weight loss and handgrip muscle strength improvement among MBS candidates. Adding a smartphone-based platform to the MBS nutritional preparation process had a significant effect on PA initiation, which may potentially have long-term positive health effects, but only modest or no effects on other measured parameters. Nevertheless, participants rated the smartphone-based platform as easy to use and having a meaningful added value, indicating the high feasibility and potential advantages of integrating mobile technology-based modules into the MBS nutritional preparation process. Larger intervention studies are needed to further investigate the benefits of integrating eHealth strategies into the MBS nutritional and lifestyle preparation process.

## Supplementary Information

Below is the link to the electronic supplementary material.

Supplementary file1 (PNG 110 KB)

## Data Availability

The data that support this study are available from the corresponding author, upon reasonable request.

## Abbreviations

BMI: Body mass index
MBS: Metabolic bariatric surgery
OAGB: One anastomosis gastric bypass
PA: Physical activity
RCT: Randomized controlled trial
RD: Registered dietitian
RYGB: Roux-en-Y gastric bypass
SD: Standard deviation
SG: Sleeve gastrectomy
VAS: Visual analog scale
WC: Waist circumference
