# Supplementary figures and images for: The Effect of Adding a Smartphone-Based Platform to the Metabolic Bariatric Surgery Nutritional Preparation Process: A Randomized Controlled Trial
Source: Obes Surg. 2025 Mar 12;35(4):1285–96. doi: 10.1007/s11695-025-07732-9 (PMC11976839; doi:10.1007/s11695-025-07732-9)

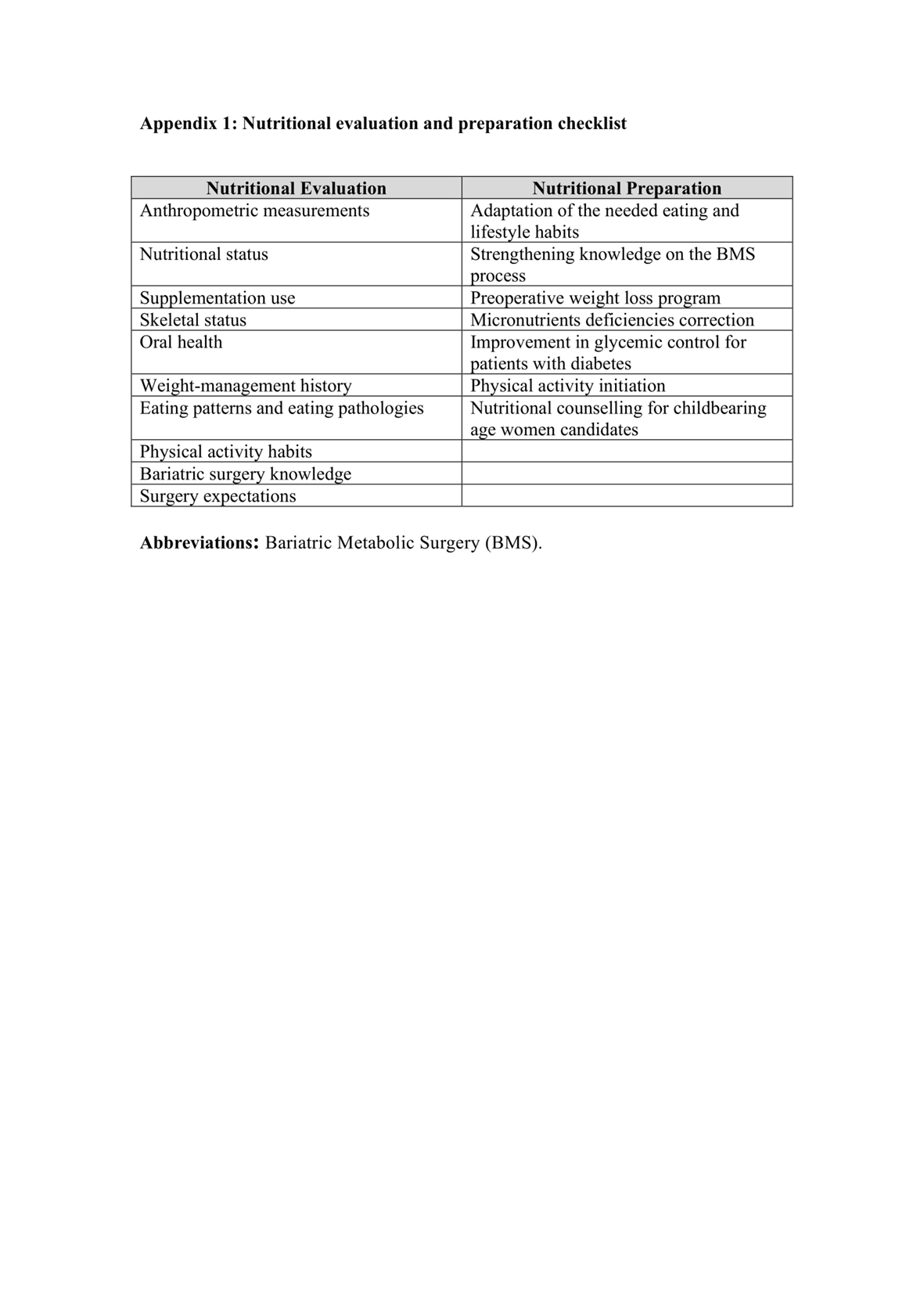

Supplement: Supplementary file 1 — Supplementary file1 (PNG 110 KB) [file 11695_2025_7732_MOESM1_ESM.png]
